# Bioefficacy of Some Egyptian Aromatic Plants on *Culex pipiens* (Diptera: Culicidae) Adults and Larvae

**Published:** 2017-03-14

**Authors:** Elham A El Zayyat, Mohammed I Soliman, Noha A Elleboudy, Shaimaa E Ofaa

**Affiliations:** 1 Parasitology Department, Faculty of Medicine, Ain-Shams University, Cairo, Egypt; 2 Research Institute of Medical Entomology, Dokki, Egypt

**Keywords:** *Culex pipiens*, *Anethum graveolens*, *Ocimum basilicum*, *Thymus vulgaris*, Insecticides

## Abstract

**Background:**

Protecting the environment from chemical hazards of synthetic insecticides along with offering of new breeding areas for vectors by urbanization indicate the trial of natural insecticides.

**Methods:**

The acetone extracts of *Anethum graveolens*, *Ocimum basilicum* and *Thymus vulgaris* were tested for their insecticidal effect on *Culex pipiens* adults and larvae in different concentrations depending on the technique used.

**Results:**

The extracts were significantly effective in all models used with basil being the best in all tested three techniques (LC_50_= 0.064) in larval feeding, (LC_50_= 0.330) in CDC bottle assay and (LC_50_= 13.148) in adults feeding (P< 0.05).

**Conclusion:**

The results recommend the eco-friendly studied extracts as candidates for controlling *Cx. pipiens* the lymphatic filariasis vector.

## Introduction

Climatic changes can cause expanding the range of geographical distribution and dynamics of vector-borne diseases. Global warming and changing sea level may lead to increase in transmission intensity and extending spatial distributions of these diseases (Hongoh et al. 2012) by influencing the development rate, longevity of insects and vector reproduction rates, creating new challenges in these insects control in areas where they were never recorded before. Anthropogenic factors with changes in landscaping, rising in housing and settlements, extensive tropical urbanization, colonization, increase in environmental pollution and changing lifestyle patterns offer new breeding areas for vectors development (Tatem et al. 2006, Weaver and Reisen 2010, Carvalho et al. 2014).

Insecticides overuse led to several ecological drawbacks over the past years. The toxicity, development of resistance phenomenon and the residual effects of these insecticides are the main concern of scientists. The urging need for developing environmental friendly insecticides is rising (Kebede et al. 2010). Several studies on botanicals potential as insecticides are ongoing with the rise of green insecticides concepts and awareness of these safe, specific, biodegradable, eco-compatible components (Park et al. 2005, Khater and Shalaby 2008, Kumar et al. 2012).

Mosquitoes are a major public health threat as they play a vital role in transmitting serious human diseases to hundreds million people annually (Akinkurolere et al. 2011). Mosquitoes were reported up to 2551–2528 BC also the invention of mosquito nets were attributed to the ancient Egyptians as Herodotus reported in 445–440 B.C. (Arnold 1995). *Culex pipiens* is a worldwide mosquito causing nuisance and transmitting many dangerous diseases as West Nile virus, St Louis encephalitis virus, filarial worms and avian malaria (Farajollahi et al. 2011). With the emergence of *Cx. pipiens* resistance to many insecticides, control is becoming more difficult providing a flourishing chance to these diseases (Zayed et al. 2006, Knio et al. 2008)

Essential oils are volatile oils with strong aroma giving distinctive odor, flavor and scent to a plant. They are secondary metabolites characterized by being complex mixtures of organic compounds (Okunowo et al. 2013). Usage of essential oils as a whole usually have higher efficacy than using its separate metabolites (Mourey and Canillac 2002, Bassole and Juliani 2012) as synergism of all compounds of the essential oil seems to be important (Zoubiri and Baaliouamer 2011). Despite being safe for domestic insect control strategies, few products based on plant essential oils are commercially available (Koul et al. 2008, Sutthanont et al. 2010).

From the ancient Pharaohs, Egyptians were familiar with aromatic plants and included their use in religion, cosmetics, embalming and medicinal purposes. In addition, the first record of essential oil distillation was from Egypt (Fakhry 2004). Egypt has a variety of flora, among which *Anethum graveolens* (Dill), *Ocimum basilicum* (*Basil*) and *Thymus vulgaris* (Thyme) were chosen to conduct this study being available in local market, cheap and widely used in traditional medicine.

This study aimed at determining the insecticidal effect of three indigenous plant extracts, commercially available in Egypt for domestic use, to control *Culex pipiens* adults and larvae.

## Materials and Methods

### Collection of plants materials

*Anethum graveolens*, *Oc. basilicum* and *Th. vulgaris* were purchased from local market, washed with distilled water to remove dust particles and identified by Department of Botany, Faculty of Science, Ain-Shams University staff members.

### Preparation of plant extracts

The whole plants including leaves and stems were dried for 7V10 days in the shade at 27–37 °C. The dried plants were powdered using a commercial electric blender then soaked in acetone 99% (Biochem. Company, Egypt) at a ratio of 1:4 for three days. Then the extracts were sieved and filtered through a Buchner funnel with sterile Whatman filter paper number one, acetone was evaporated using Rotator evaporator apparatus (Hs-2005s, Jisico Co., Ltd. Korea) in Central laboratories, Faculty of Science, Ain-Shams University, Egypt. The extracts were concentrated under reduced pressure 22–26mm Hg at 45 °C and the crude extracts residue were kept in dark bottles, labeled and preserved in the refrigerator at 4 °C until further use (Akinkurolere et al. 2011).

### Insects rearing

Laboratory reared colony of *Cx. pipiens* free from insecticides and pathogens obtained from Mosquitoes Research Department, Research Institute of Medical Entomology, Ministry of Health and Population, Dokki, Giza was maintained starting from egg rafts. Larvae used in the tests were reared in a plastic cup containing dechlorinated water, under the standard conditions of 28±2 °C temperature and 70±5% RH and L12/D12 photoperiod and fed daily on tetramine (tropical fish food) until the larvae transformed into the pupal stage. The larvae were reared until adults in insectaries. Adults were maintained on a 10% sugar solution and females were allowed to feed periodically on guinea pig blood for 2–3h every two days to obtain protein used principally for egg production (Kovendan et al. 2012).

### Larvicidal bioassay

The assay was done on healthy late third instar larvae, three replicates for each test were used. Larvae were collected with a pasture pipette, placed on a filter paper to remove excess water then placed in cups containing 250mL dechlorinated tap water to which different concentrations of the tested extracts were added (0.02g/L, 0.05g/L, 0.1 g/L, 0.2g/L and 0.3g/L) then readjusted according to obtained results. Three controls cups containing water were used in the presence of the larvae semi synthetic diet. All cups were covered with muslin cloth for protection. The observed mortality was recorded at the end of 72h, larvae were considered dead if there is no sign of any movement even after mild touch with a glass rod (Kumar et al. 2014).

### Adulticidal bioassay

Adaptation of CDC Bottle Bioassays technique was done to evaluate insecticidal properties of the extracts as follows: 250ml Wheaton bottles with screw lids were properly cleaned and dried then they were coated with 1ml of (0.2g/L, 0.4g/L, 0.6g/L, 0.8g/L) of the tested extracts by swirling assuring complete coating of the bottle and its cap. One ml of absolute alcohol was added to the control bottle handled as before. Forty mosquitoes, divided among four replicate bottles were introduced using an aspirator into each bottle with the tested extract concentrations (Brogdon 2014). Each bioassay included a control bottle with 10 mosquitoes. Mortality was assessed after 2h then after 24h after the mosquitoes’ introduction and mosquitoes were considered dead if they could no longer stand (Aïzoun et al. 2013). The experiment was done under normal room conditions.

Feeding toxicity was done according to Allan (2011) for *Cx. pipiens* adults using randomly selected male and female, 3–6 days after emergence, starved for 24h, and placed in cages 0.5× 0.5× 0.5m (10/cage), Baits were presented in a 1cm length of cotton wick soaked with 200*μ*l of 10% sucrose containing the tested extracts doses (10g/L, 20g/L, 40g/L) and placed on top of the mesh of each cage and removed after 2h. The insects with distended abdomens indicating ingestion of a sugar meal were transferred to a new cage (10/cage) and maintained. A cotton pad soaked with glucose only solution was used as a control for each extract. Mortality was assessed up to 72h after the treatment performance. Adults that did not respond to pin prick were be considered dead.

Percentage of mortality was corrected using of Abbott’s formula (Abbott 1925) if needed.

$$\%\;\text{Mortality}=\frac{\%\;\text{test}\;\text{mortality}-\%\;\text{control}\;\text{mortality}}{100-\%\;\text{control}\;\text{mortality}}\times 100$$

All statistical tests were performed using The SPSS version 22, 2013 (14.0, Chicago, IL, USA). A probit regression model to predict the probability of percentage of insect mortality and logarithmic concentration of the extracts was done. *Z*-scores were estimated. Pearson Goodness-of-Fit of Chi-square test to determine the applicability of the resultant regression models was done. LC_50_, LC_90_ with their 95 per cent confidence limits were determined. The Toxicity index was calculated as follows, lLC_50_ of the most effective compound ×100 divided by the lLC_50_ of the compound used and the most toxic compound was given 100 units on the toxicity index scale according to Rawi et al. (2011). The probability of error at 0.05 was considered significant, while at 0.01 and 0.001 were considered highly significant throughout the whole study.

## Results

Evaluation of insecticidal properties of three indigenous plants was done using different *An. graveolens*, *Oc. basilicum* and *Th. vulgaris* crude extracts concentrations on both larvae and adults of *Cx. pipiens* mosquito. The results revealed the effectiveness of the used extract different doses to obtain mortality and suggested the potentiality of their usage in *Cx. pipiens* control. The results of larvicidal activity of extracts by feeding technique after 72h are shown in Table 1 and 2). Adulticidal activity of the tested extracts by feeding technique after 72h is shown in Table 3 and 4 and by CDC bottle bioassay after 24 h is shown in Table 5 and 6. Plate (1) shows scanning electron microscopy of *Cx. pipiens* larva induced by basil extract after 72h while plate (2) for *Cx. pipiens* larva induced by basil extract by CDC technique after 24h.

**Table 1.** Probit analysis of larvicidal activity of the tested extracts against *Culex pipiens* by feeding technique after 72h

**Plant**	**Parameter**	**Mortality rate**	**Standard Error**	**Z**	**Sig**	**95% Confidence Interval**		**Pearson Goodness of Fit Test**	

						**Lower Bound**	**Upper Bound**	**Chi-Square**	**Sig**
**Thyme**	**Slope**	1.057	0.228	4.641	.000	0.611	1.503	0.725	0.867
	**Intercept**	1.220	0.204	5.991	.000	1.016	1.424		
**Dill**	**Slope**	1.428	0.192	7.442	.000	1.052	1.804	2.319	0.509
	**Intercept**	1.642	0.217	7.554	.000	1.424	1.859		
**Basil**	**Slope**	0.996	0.190	5.257	.000	0.625	1.368	1.589	0.662
	**Intercept**	1.187	0.211	5.615	.000	0.976	1.398		

**Table 2.** Larvicidal activity of the tested doses of extracts against *Culex pipiens* by feeding technique after 72h

**Plant**	**Concentration (g/L)**	**Log Concentration**	**Observed Responses**	**Expected Responses**	**Residual**	**Probability**
**Thyme**	0.02	−1.699	15	15.935	−0.935	0.398
	0.05	−1.301	24	22.590	1.410	0.565
	0.1	−1.000	28	27.392	0.608	0.685
	0.2	−0.699	30	31.518	−1.518	0.788
	0.3	−0.523	34	33.512	0.488	0.838
**Dill**	0.02	−1.699	14	13.000	1.000	0.217
	0.05	−1.301	26	24.883	1.117	0.415
	0.1	−1.000	30	35.091	−5.091	0.585
	0.2	−0.699	46	44.413	1.587	0.740
	0.3	−0.523	50	48.883	1.117	0.815
**Basil**	0.02	−1.699	12	12.263	−0.263	0.307
	0.05	−1.301	17	18.262	−1.262	0.457
	0.1	−1.000	26	23.026	2.974	0.576
	0.2	−0.699	28	29.893	−1.893	0.747
	0.3	−0.523	33	32.500	0.500	0.812

**Table 3.** Probit analysis of adulticidal activity of the tested extracts against *Culex pipiens* by feeding technique after 72h

**Plant**	**Parameter**	**Mortality rate**	**Standard Error**	**Z**	**Sig**	**95% Confidence Interval**		**Pearson Goodness of Fit Test**	

						**Lower Bound**	**Upper Bound**	**Chi-Square**	**Sig**
**Thyme**	**Slope**	1.903	0.571	3.334	.001	0.785	3.022	0.215	0.643
	**Intercept**	−2.475	0.754	3.281	.001	−3.229	−1.721		
**Dill**	**Slope**	3.066	0.636	4.823	.000	1.820	4.311	0.042	0.837
	**Intercept**	−3.772	0.824	4.579	.000	−4.595	−2.948		
**Basil**	**Slope**	1.496	0.568	2.632	.008	0.382	2.610	1.063	0.303
	**Intercept**	−1.674	0.741	−2.26	.024	−2.415	−0.933		

**Table 4.** Adulticidal activity of the tested doses of extracts against *Culex pipiens* by feeding technique after 72h

**Plant**	**Concentration (g/L)**	**Log Concentration**	**Observed Responses**	**Expected Responses**	**Residual**	**Probability**
**Thyme**	10	1.000	9	8.514	0.486	0.284
	20	1.301	14	15.016	−1.016	0.501
	40	1.602	22	21.514	0.486	0.717
**Dill**	10	1.000	7	7.201	−0.201	0.240
	20	1.301	18	17.573	0.427	0.586
	40	1.602	26	26.183	−0.183	0.873
**Basil**	10	1.000	14	12.883	1.117	0.429
	20	1.301	16	18.221	−2.221	0.607
	40	1.602	24	22.953	1.047	0.765

**Table 5.** Probit analysis of adulticidal activity of the tested extracts by CDC bottle bioassay after 24h

**Plant**	**Parameter**	**Mortality rate**	**Standard Error**	**Z**	**Sig**	**95% Confidence Interval**		**Pearson Goodness-of-Fit Test**	

						**Lower Bound**	**Upper Bound**	**Chi-Square**	**Sig**
**Thyme**	**Slope**	2.750	0.570	4.825	.000	1.633	3.868	3.383	0.184
	**Intercept**	0.988	0.233	4.246	.000	0.755	1.221		
**Dill**	**Slope**	2.964	0.595	4.984	.000	1.799	4.130	2.465	0.292
	**Intercept**	0.906	0.232	3.911	.000	0.674	1.138		
**Basil**	**Slope**	3.247	0.592	5.480	.000	2.085	4.408	1.011	0.603
	**Intercept**	1.561	0.262	5.967	.000	1.300	1.823		

**Table 6.** Adulticidal activity of the tested doses of extracts against *Culex pipiens* by CDC bottle bioassay after 24h

**Plant**	**Concentration (g/L)**	**Log Concentration**	**Observed Responses**	**Expected Responses**	**Residual**	**Probability**
**Thyme**	0.2	−0.699	7	5.252	1.748	0.175
	0.4	−0.398	10	13.729	−3.729	0.458
	0.6	−0.222	19	19.418	−0.418	0.647
	0.8	−0.097	25	22.941	2.059	0.765
**Dill**	0.2	−0.699	5	3.654	1.346	0.122
	0.4	−0.398	9	11.765	−2.765	0.392
	0.6	−0.222	17	17.941	−0.941	0.598
	0.8	−0.097	24	21.958	2.042	0.732
**Basil**	0.2	−0.699	8	7.182	0.818	0.239
	0.4	−0.398	16	18.183	−2.183	0.606
	0.6	−0.222	25	23.994	1.006	0.800
	0.8	−0.097	27	26.811	0.189	0.894

## Discussion

Natural insecticides are of growing interest worldwide being eco-friendly as with climatic changes and urbanization offering new breeding areas for vectors, it is becoming mandatory to meet the needs for screening of plants potential insecticidal effect. Plants phytochemical compounds have demonstrated a promising potential for insecticidal activity. The toxicological safe nature of most essential oils and its availability in developing countries with huge biodiversity and complex mixtures of its constituents resulting in more slowly development of resistance may ultimately have their greatest impact in future insects control programs (Koul et al. 2008).

The usage of plant crude extracts as a whole besides being less expensive, it advances the synergistically of its active compounds complex mixtures providing greater bioactivity compared to its purified individual constituents (Cavalcanti et al. 2004). Several studies reported the botanical insecticidal effect on *Culex* mosquito with variation depending on geographical origin of the plant, the plant parts processed, type of solvent used and the *Culex* species tested (Jeyabalan et al. 2003). The mortality effect of plant extracts evaluated in this study varied according to the plant, concentration of the extract and the used technique.

Highly significant linear predictions from the resultant probit models were obtained as shown by z-scores. There was a non-significant difference on comparing the observed and expected response by chi-square goodness of fitness test (Tables 1, 3 and 5) so assumptions made by this study were reasonable and the choice of models were appropriate and the frequency counts distributed identically across different populations.

Dose response relationship of the tested extracts showed increase in the mortality rate with increasing the tested doses of extracts with non-significant difference between the observed value of mortality and the predicted value, the residual (Tables 2, 4 and 6) indicating the appropriateness of the used models. The mechanism of toxicity of essential oils on insects needs further study to be clarified. Comparing these results to other studies on same extracts may vary in results as variation of plant essential oil composition according to its indigenous origin, target mosquito species tested, method tested, different exposure times and extract concentrations used (Kumar et al. 2011).

In larvicidal assay of the tested extracts by feeding technique after 72h, basil was highly effective (LC_50_= 0.064) followed by thyme (LC_50_= 0.070) and dill (LC_50_= 0.071). Concerning adulticidal activity of the tested extracts by feeding technique after 72h basil was highly effective (LC_50_= 13.148) followed by dill (LC_50_= 16.996) and thyme (LC_50_= 19.967) and regarding adulticidal activity of the tested extracts by CDC bottle bioassay, no mortality recorded after 2h but after 24h basil was highly effective (LC_50_= 0.330) followed by thyme (LC_50_= 0.437) and dill (LC_50_= 0.495) according to the lowest LC50 and in return the highest toxicity indices. So *Ocimum basilicum* was the most effective extract tested on *Cx. pipiens* larvae and adults in accordance to other studies as Aarthi and Murugan (2010), Belong et al. (2013) and Govindarajan et al. (2013).

## Conclusion

The integrated usage of indigenous *An. graveolens*, *Oc. basilicum* and *Th. vulgaris* in control of *Cx. pipiens* would probably help in reducing the magnitude of vector borne diseases. They provide promising results as insecticides besides being cheap, available and easily handled. Further studies on the mechanism of killing, effect on insect development, physiology and metabolism as well as field trials are recommended.
